# Predicting factors for survival of breast cancer patients using machine learning techniques

**DOI:** 10.1186/s12911-019-0801-4

**Published:** 2019-03-22

**Authors:** Mogana Darshini Ganggayah, Nur Aishah Taib, Yip Cheng Har, Pietro Lio, Sarinder Kaur Dhillon

**Affiliations:** 1 Data Science and Bioinformatics Laboratory, Institute of Biological Sciences, Faculty of Science, University of Malaya, 50603 Kuala Lumpur, Malaysia; 2 Department of Surgery, Faculty of Medicine, University of Malaya, 50603 Kuala Lumpur, Malaysia; 3 Department of Computer Science and Technology, University of Cambridge, 15 JJ Thomson Avenue, Cambridge, CB3 0FD England

**Keywords:** Data science, Machine learning, Factors influencing survival of breast cancer, Random forest, Decision tree

## Abstract

**Background:**

Breast cancer is one of the most common diseases in women worldwide. Many studies have been conducted to predict the survival indicators, however most of these analyses were predominantly performed using basic statistical methods. As an alternative, this study used machine learning techniques to build models for detecting and visualising significant prognostic indicators of breast cancer survival rate.

**Methods:**

A large hospital-based breast cancer dataset retrieved from the University Malaya Medical Centre, Kuala Lumpur, Malaysia (*n* = 8066) with diagnosis information between 1993 and 2016 was used in this study. The dataset contained 23 predictor variables and one dependent variable, which referred to the survival status of the patients (alive or dead). In determining the significant prognostic factors of breast cancer survival rate, prediction models were built using decision tree, random forest, neural networks, extreme boost, logistic regression, and support vector machine. Next, the dataset was clustered based on the receptor status of breast cancer patients identified via immunohistochemistry to perform advanced modelling using random forest. Subsequently, the important variables were ranked via variable selection methods in random forest. Finally, decision trees were built and validation was performed using survival analysis.

**Results:**

In terms of both model accuracy and calibration measure, all algorithms produced close outcomes, with the lowest obtained from decision tree (accuracy = 79.8%) and the highest from random forest (accuracy = 82.7%). The important variables identified in this study were cancer stage classification, tumour size, number of total axillary lymph nodes removed, number of positive lymph nodes, types of primary treatment, and methods of diagnosis.

**Conclusion:**

Interestingly the various machine learning algorithms used in this study yielded close accuracy hence these methods could be used as alternative predictive tools in the breast cancer survival studies, particularly in the Asian region. The important prognostic factors influencing survival rate of breast cancer identified in this study, which were validated by survival curves, are useful and could be translated into decision support tools in the medical domain.

## Background

Breast cancer appears to be the most common cancer type suffered by women across the globe, which stands after lung cancer amidst developed nations [1–3]. In Malaysia, 50–60% of breast cancer cases are detected at late stages, hence the survival of the patients is one of the lowest in the region [4–6]. Accordingly, it is necessary to determine the various factors that influence the survival rate among breast cancer patients.

Previously clinicians have used basic software programs, such as Microsoft Excel, SPSS, and STATA [7–9], in analysing factors influencing breast cancer survival rate. These conventional statistical methods are not really adaptable in identifying new variables as well as generating creative and integrative visualisations [10]. The drawback of these conventional statistical analyses has led to the wide usage of various machine learning (ML) approaches such as decision tree (DT), random forest (RF), neural networks, extreme boost, logistic regression and support vector machine (SVM) in this field [11–18]. Decision tree is a supervised learning algorithm, which illustrates the results in an easily interpretable tree structure where visualisation is an important factor in analysing large number of data [19–22]. Random forest (*Breiman’s* algorithm), which is a derivation of DT, is able to work in both supervised and unsupervised mode, handling continuous and categorical data in classification or regression tasks [23, 24]. Neural networks are complex and have often been regarded as black box, which perform modelling by training from the data that have a known outcome and optimising weights for better prediction in situations with unknown outcome [25, 26]. Extreme boost is an ensemble of classification and regression tree, which is parallelizable, is able to produce effective prediction accuracy, an easy to use algorithm and has outperformed other algorithms in several machine learning competitions [27]. Logistic regression follows Gaussian distribution and handles all types of variables such as continuous, discrete and dichotomous, which does not need a normality assumption [28, 29]. Support vector machine is used for supervised classification and it works by identifying the optimal decision boundary that separates data points from varying groups, and then, predicting the class of new observations based on this separation boundary [30].

Even though machine learning models for breast cancer were previously built and analysed, the factors may vary based on different locations, lifestyle and available data. Thus, we found that it is necessary to build models for the Malaysian context to determine the factors influencing survival rate of breast cancer patients. It is also very useful to perform variable selection using machine learning methods in the medical domain where traditional statistical methods have been a preference among the clinicians [31, 32].

The aim of this study is to identify the important prognostic factors influencing survival rate of breast cancer patients in the Asian setting using standard machine learning techniques to create interpretable prognostic models.

## Methods

This study adhered to the data science life cycle methodology to perform analysis on a set of data pertaining to breast cancer patients as elaborated by Wickham and Grolemund [33]. All the methods except calibration analysis were performed using R (version 3.5.1) [34] with default parameters. R is a popular open-source statistical software program [35]. Calibration analysis was performed using Python3 [36].

### Data collection

A large hospital-based dataset that consists of 8942 breast cancer patients’ data was obtained from the Breast Cancer Registry of University Malaya Medical Centre (UMMC), Kuala Lumpur, Malaysia. This dataset was already de-identified in compliance with Laws of Malaysia Act 709, Personal Data Protection Act 2010. Initially, 113 unorganised variables were found in the dataset. A discussion with several clinicians in UMMC led to the removal of 89 unnecessary variables that were considered as less significant prognostic factors for breast cancer survival. Data pre-processing was carried out by importing the dataset with remaining 24 variables in comma-separated format followed by removing the rows with substantial missing values by using the *na.omit* function. The clean dataset of 8066 (‘all data’) patients’ records contained 23 independent/predictor variables and 1 dependent/categorical variable, which reflected the survival status of the patients (alive/dead). All variables, along with their descriptions, values, and proportion of each value, are listed in Table 1 (nominal variables) and Table 2 (numerical variables). Next, the data was clustered based on the receptor status of breast cancer patients identified via immunohistochemistry (IHC). Receptor status was selected to cluster the dataset, mainly because it was used to classify the records obtained from breast cancer patients for further analysis in studies associated to survival prediction [8, 9, 37, 38]. Three clusters were segregated based on Estrogen receptor (ER) status, Progesterone receptor (PR) status, and c-er-b2 status, as listed in Table 3.

**Table 1:** Description of nominal variables in the breast cancer dataset

Nominal variable	Name	Description	Value	Proportion (%)
V2	Marital status	The marital status of the patients	Married	81.6
			Not married	18.4
V3	Menopausal status	The way of menopausal encountered by the patients	Natural menopause	50.6
			Pre-menopause	42.8
			Surgical menopause	6.6
V4	Presence of family history	Presence of breast cancer in family history	Yes	81.2
			No	18.8
V5	Race	Ethnicity	Chinese	68.4
			Malay	19.7
			Indian	11.9
V6	Method of diagnosis	The method used by clinicians to confirm the diagnosis of breast cancer	Excision	20.8
			FNAC (Fine Needle Aspiration Cytology)	24.5
			Imaging only	0.5
			Trucut	54.2
V7	Classification of breast cancer	Invasive cancer is a type of malignant cell, can spread to other parts of body, called metastasized. In situ cancer is recognizable as malignant cell, but have not begun to act as malignant fashion, does not spread and does not go past the breast	Invasive	95.3
			Insitu	4.7
V8	Laterality	The laterality of breast diagnosed with cancer	Left	45.5
			Right	49.5
			Bilateral	1.3
			Unilateral	3.7
V9	Cancer stage classification	Stage 0	Pre-cancer	4.6
		Stage 1, Stage 2, Stage 3	Curable cancer	84.2
		Stage 4	Metastatic cancer	11.2
V10	Grade of differentiation in tumour	Description of a tumour based on how abnormal the tumour cells and the tumour tissue look under a microscope. It is an indicator of how quickly a tumour is likely to grow and spread. G1 is poor, G2 is moderate, G3 and G4 are good differentiation described in this dataset.	Good	32.9
			Moderate	37.1
			Poor	30.0
V12	Eestrogen receptor (ER) status	Normal breast cells and some breast cancer cells have receptors that attach to the hormone Estrogen and depend on this hormone to grow. Breast cancers that have this hormone are called ER-positive.	Positive	58.9
			Negative	41.1
V13	Progesterone receptor (PR) status	Normal breast cells and some breast cancer cells have receptors that attach to the hormone progesterone and depend on this hormone to grow. Breast cancers that have this hormone are called PR-positive.	Positive	46.0
			Negative	54.0
V14	c-er-b2 status	c-er-b2 is a gene that produces a protein which acts as a receptor on the surface of the cancer cells. It is a proto-oncogene located on chromosome 17. This gene is amplified and thus the protein (HER-2) is over-expressed in around 20 to 25% of invasive breast cancers.	Positive	24.1
			Negative	65.4
			Equivocal	10.5
V15	Primary treatment type	The type of treatment underwent by the patients as their initial or first treatment.	Chemotherapy	12.6
			Hormone Therapy	3.4
			Surgery	77.8
			None	6.2
V16	Surgery status	The status of the patients weather they have been treated with surgery or not.	Surgery done	85.5
			No surgery	14.5
V17	Type of surgery	The type of surgery done to the cancer patients. The type of surgery depends on the cancer stage and tumour size.	Breast Conserving surgery	24.3
			Mastectomy	61.1
			No surgery	14.6
V18	Method of axillary lymph node dissection	Yes if it is done. The methods used to remove the axillary lymph nodes from the breast (SLNB, SLNB to AC). None, if it is not done.	Yes	70.6
			SLNB (Sentinel lymph node biopsy)	6.7
			SLNB to AC (Axillary clearance)	0.4
			None	22.3
V19	Radiotherapy	The status of the patients weather they have been treated with radiotherapy or not.	Radiotherapy	49.4
			No Radiotherapy	50.6
V20	Chemotherapy	The status of the patients weather they have been treated with chemotherapy or not.	Chemotherapy	54.3
			No chemotherapy	45.7
V21	Hormonal therapy	The status of the patients weather they have been treated with hormone therapy or not.	Hormonal therapy	54.9
			No hormonal therapy	45.1
V24	Status	The survival status of the patients.	Alive	69.6
			Dead	30.4

**Table 2:** Description of numerical variables in the breast cancer clinical dataset

Numerical variable	Name	Description	Minimum	Mean	Maximum
V1	Age at diagnosis	Age of the patients when they are diagnosed with breast cancer	0	50	92
V11	Tumour size (cm)	The size of tumour (cm)	0	3.2	30
V22	Total axillary lymph nodes removed	The number of total axillary lymph nodes removed for examination	0	13	45
V23	Number of positive lymph nodes	The number of lymph nodes identified as cancerous	0	3	19

**Table 3:** Clusters of breast cancer data based on receptor status

No	Cluster	Estrogen receptor (ER)	Progesterone receptor (PR)	c-er-b2 status	Samples
1	Hormone Receptor Sensitive (HRS)	+	+	+/−	3520
2	c-er-b2 over-expressed	–	–	+	966
3	Basal/Triple Negative Breast Cancer (TNBC)	–	–	–	1975

The total number of samples in the three clusters was 6461 because not all patients fell under the respective receptor groups of Hormone Receptor Sensitive (HRS), c-er-b2 over-expressed, and Triple Negative Breast Cancer (TNBC). However, the number of samples for ‘all data’ remained as 8066.

### Model evaluation

In the first step, modelling was performed on the whole dataset (‘all data’) with 8066 records and 23 predictors of survival rate. The quality of data was compared using six algorithms: decision tree (*rpart*) [39], random forest (*randomForest*) [40], neural networks (*neuralnet*) [41], extreme boost (*xgboost*) [42], logistic regression (*glm*), and support vector machine (*e1071*) [43]. The dataset was then split into a training set (70%; 5646 records) and a testing set (30%, 2420 records) for the model evaluation using all the algorithms. Each model was assessed with accuracy, sensitivity, specificity, precision, Matthew correlation coefficient, area under the receiver operating characteristic curve (AUC), precision and recall curve, and finally, calibration curve.

- *Decision tree*
  This study employed the rpart package, which implemented the classification and regression tree (CART) function to build DT for prediction and evaluation of the ‘all data’. This function processed the input and yielded the model accuracy and an optimal tree as the end-result. The DT contained a root node at the top of the tree to signify the most important variable, followed by decision nodes and terminal nodes with percentages of classification. We selected DT as one of the algorithms to evaluate the data as it is known to handle various types of data [19–22].
- *Random forest*
  Random forest segregated the dataset into 70% of training data automatically for learning. In this algorithm we did not manually split the data into training and testing sets prior to prediction, as required for other algorithms. Each tree was grown independently and the final prediction using test dataset yielded accurate prediction using mean value. Hence, it was able to achieve best-in-class performance with respect to low generalisation error. As for this study, the default number of trees (ntree = 500) in RF was employed to assess the model accuracy. RF appeared to be a suitable classifier to evaluate the model as the breast cancer dataset used in this study contained both continuous and categorical variables, which classified the survival status as either alive or dead.
- *Neural networks*
  This study applied the multi-layer-perceptron based artificial neural networks (MLP-ANN); a feed-forward and supervised learning technique composed of input, hidden, and output layers [44]. The input values (23 predictor variables) were presented to the perceptron, and if the predicted output was similar to the desired output, the performance was considered satisfactory and no weight was changed, portraying exceptional accuracy. The neural network was selected in this study to perform model evaluation as it worked well when data volatility was very high. The feed forward neural network was selected to avoid complications from feedback networks that introduce loop in the network.
- *Extreme boost*
  In this study, extreme boost modelling was performed by converting the testing and training data into matrix as xgboost only supports matrix for model evaluation. This algorithm performs better when the training data is large with numeric or a combination of numeric and categorical data because it has the capability to handle complicated and diverse types of variables [45]. This study presents a combination of numeric and categorical data, as described in Tables 1 and 2.
- *Logistic regression*
  The Gaussian distribution in logistic regression possesses odds ratio, where the log odds of the dependent variable (survival status) were modelled as the linear combination of the independent variables (23 factors that influenced survival status). As logistic regression is appropriate for dichotomous (binary) dependent variable, the survival status of the patients from alive/dead was replaced with 1/0 in the dataset when performing model evaluation using logistic regression. We chose logistic regression as one of the algorithms to evaluate the model accuracy as the dependent variable is a survival status (alive/dead), which can be evaluated using binary values.
- *Support vector machine*
  Support vector machine managed the problem of quadratic optimization in this dataset by creating optimum separating borders between data. Support vector coordinates of an individual observation or the variable supported both linear and non-linear class boundaries. SVM was selected as one of the algorithms to examine the model performance because it captured inherent characteristics of data better.
- *Calibration analysis*
  A calibration analysis was performed in this study using the scikit learn module in Python3. The packages used were RandomforestClassifier, DecisionTreeClassifier, MLPClassifer, GradientBoostingClassifier, LogisticRegression, and LineraSVC in order to validate the reliability of the dataset for ‘all data’.

### Random forest advanced modelling

RF seems to be the preferred algorithm in most clinical studies [23, 24]. It has been reported to generate one of the best accuracies and is superior over other techniques in terms of its ability in handling highly non-linear data and a large number of features, agile in terms of noise in data, and simpler to tune than other ensemble learning algorithms [46]. RF algorithm is composed of several features, such as its effective method in estimating missing value, its Weighted Random Forest (WRF) for balancing errors in imbalanced data, and its estimation on the importance of variables used for classification [13]. Thus, advanced modelling was performed using RF by choosing the best *ntree* (number of trees) value for ‘all data’ (*n* = 8066). Next, the best *ntree* value was used to evaluate the clusters of data based on receptor status. This algorithm worked in two stages: the first was to create RF, and the second to make prediction from the created RF. The first stage consisted of five steps: (a) “X” features were selected randomly from a total of 23 features where X < 23, (b) among the X features, the node “n” was calculated using the best split point, (c) the node was split into daughter nodes using the best split, (d) steps (a - c) were repeated until “Y” number of nodes was attained, and lastly, (e) the forest was built by repeating steps (a – d) for “n” number of times to create “n” number of trees. Next, the second stage consisted of three steps: (a) test features and the rules of each randomly created DT were used to predict the outcomes and to store the predicted outcomes (Alive/Dead), (b) the votes for each predicted target were calculated, and lastly, (c) the highly voted predicted target was considered as the final prediction from the RF algorithm. The best *ntree* value with the least Out of Bag (OOB) error was determined based on the index of the minimum element of the vector.

### Variable selection

The next step was to select variables for further modelling and visualisation. Variable selection is a pertinent procedure in prediction and decision analysis, especially when dealing with clinical data. Variable selection is important to produce a better predictive model only by using integral variables, instead of predicting survival using all available variables, which can generate complicated and non-readable outputs and visualisations. In this study, variable selection was performed by adopting the threshold-based pre-selection method. ‘All data’ and clustered datasets were used for selection of variables. Variable selection was performed using two packages: *VSURF* [47], and *randomForestExplainer* [48]. The variable selection analysis using both these packages was first applied to ‘all data’, and then, to the three data clusters (‘HRS’, ‘c-er-b2 over-expressed’, and ‘TNBC’). A comparison was made between the important prognostic factors that influenced breast cancer survival rate, as determined by these two packages.

- *VSURF*
  *VSURF*, which is defined as variable selection using random forest, was implemented to perform model evaluation with a default *ntree* of 2000 [24]. It consisted of two steps: preliminary elimination and ranking, as well as variable selection.
  In the first step, all 24 variables in the dataset were ranked by sorting the variable importance (VI) (averaged over typically 50 RF runs) in a descending order, following the steps described in Robin et al. [24]. In the second step, there are two main processes, which are interpretation and prediction, as described by Robin et al. [24]. As for interpretation, a nested collection of RF models involving the k first variables (k = 1 to 24) was constructed and the variables involved in the model that led to the smallest OOB error were selected for interpretation. Next, the OOB error rates of RF (averaged typically over 25 runs) of the nested models were computed starting from the most important variable, and ending with the other important variables previously kept. Ideally, the variables of the model that led to the smallest OOB error were selected. As for prediction, starting with the ordered variables retained for interpretation, an ascending sequence of RF models was constructed by invoking and testing the variables in step-wise manner. The variables of the last model were selected. In precise, the sequence of the variables was determined by adhering to a rule: a variable was added only if the decreased error was larger than the threshold. The decrease in OOB error was significantly greater than the average variation obtained by adding noisy variables. The threshold was set to the mean of the absolute values of the first order that differentiated OOB errors in the model from the 24 variables, where the threshold value selected in this study was 0.01 (VI mean).
- *Random Forest Explainer*
  In the *randomForestExplainer* package, various variable importance measures were calculated and visualised in different ways to obtain an idea on how their importance changed based on the dataset. The steps that determined the important variables using *randomForestExplainer* were:
  Step 1: The data were trained with *randomForest* classifier, *ntree* = 470.
  Step 2: The created forest was passed to the function *min_depth_distribution* to obtain the distribution of minimal depth.
  Step 3: Ten important variables were plotted with the distribution of minimal depth.
  Step 4: Multi-way importance was plotted with mean squared error after permutation and increase in the node purity index (y-axis).

### Decision t*ree*

Prediction of survival rate from the important prognostic factors of breast cancer had been carried out with DT analysis by deploying *rpart* package. Four DTs were plotted using important variables, which were identified in the variable selection process, for each cluster.

### Survival analysis

Survival analysis in medicine is known to deal with occurrence of an event of interest, such as alive, death or recurrence. In this study, the years of survival for the deceased were calculated by subtracting the date of diagnosis from the date of death, and for the alive, by subtracting the date of diagnosis from the date of last contact. *Survival* [49] package was used to plot the survival curve for each important variable identified in the variable selection. In this package, the object was created by *Surv()* function, which is the basic survival analysis data structure in R, composed of the failure time and censoring information, whereas the *survfit* function computed the Kaplan-Meier estimator.

## Results

### Model evaluation

**Fig. 1:**
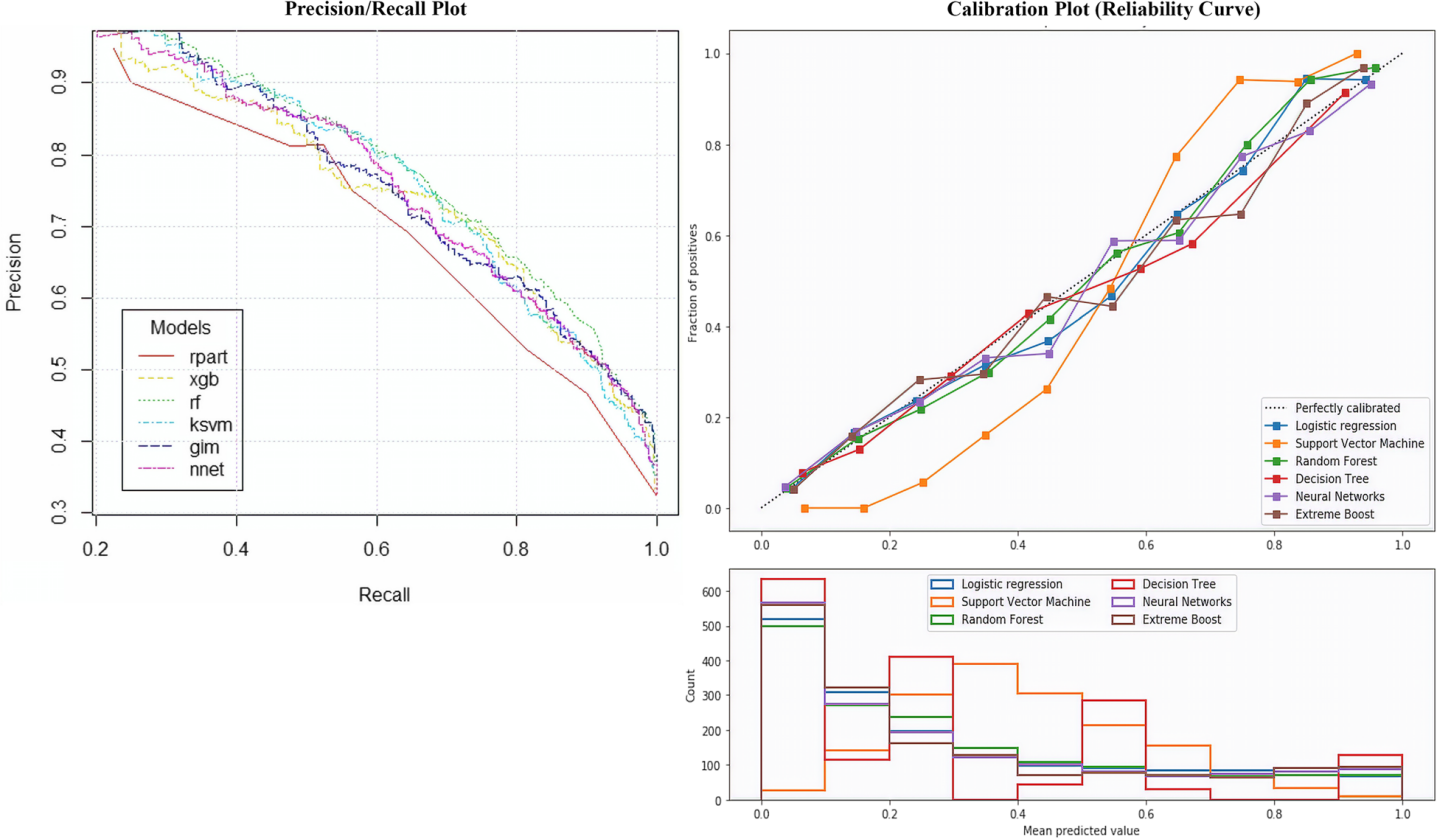
Precision and recall plot and calibration curves of all algorithms

Model accuracies of six algorithms on the samples of breast cancer data prior to clustering (‘all data’; *n* = 8066) are displayed in Table 4, while the precision-and-recall plot and the calibration measure of all the algorithms are presented in Fig. 1.

**Table 4:** Model accuracy of six algorithms. Random forest yielded slightly better accuracy using ‘all data’

No	Algorithm	Accuracy (%)	Sensitivity	Specificity	AUC	Precision	Matthews correlation coefficient
1	Decision tree	79.80	0.82	0.75	0.72	0.91	0.52
2	Random forest	82.70	0.83	0.81	0.86	0.93	0.59
3	Neural networks	82.00	0.83	0.79	0.84	0.93	0.58
4	Extreme boost	81.70	0.84	0.75	0.87	0.89	0.57
5	Logistic regression	81.10	0.82	0.78	0.85	0.92	0.55
6	Support vector machine	81.80	0.81	0.84	0.85	0.95	0.57

The RF indicated well-calibrated prediction as its curve was nearly diagonal, when compared to the other algorithms. Decision tree, neural networks, extreme boost, and logistic regression classifiers generated close calibrations, which corresponded to the model accuracy as all algorithms were close in terms of accuracy. The support vector machine classifier produced a sigmoid curve due to the margin property of hinge loss as it focused on hard samples closer to decision boundaries (the support vectors). The dataset for the prediction of breast cancer survival (‘all data’) seemed sufficiently reliable to proceed with the other steps, mainly because the calibration measures were closer to the diagonal or identity.

### Random forest advanced modelling

**Fig. 2:**
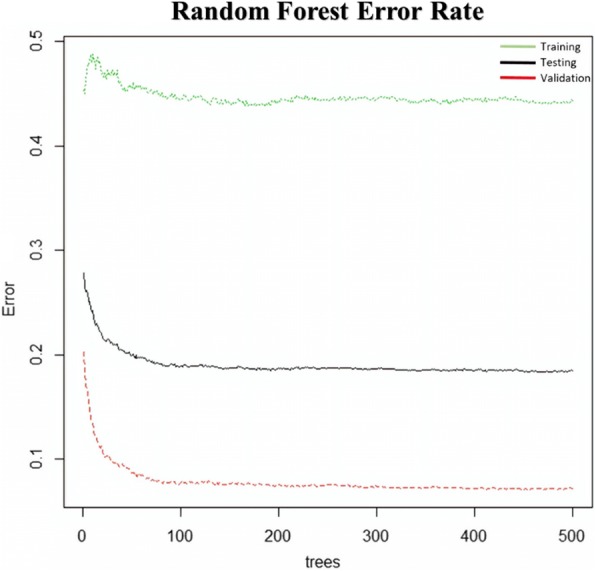
Random forest error rate

The RF algorithm produced slightly better accuracy (82.7%), in comparison to other algorithms in model evaluation. The OOB error plots of training, testing, and validation in RF are illustrated in Fig. 2. Figure 2 signifies that further modelling of ‘all data’ with RF classifier yielded the best *ntree* value of 470. The training dataset was used by the machine to learn and to fit the variables. Once the model was processed using the training dataset, predictions were made using the testing dataset. The validation dataset stopped training when the errors began increasing in order to prevent over-fitting. Hence, the training set yielded higher error rate (0.4–0.5) than that of testing dataset (0.1–0.3), followed by the validation dataset (0.0–0.2) during the final prediction. The outcomes of model evaluation for the three clusters are summarised in Table 5.

**Table 5:** Results of random forest modelling on clustered datasets based on receptor status

No	Cluster	Samples	Accuracy (%)
1	Hormone Receptor Sensitive	3520	84.00
2	c-er-b2 Over-expressed	966	77.60
3	Triple Negative Breast Cancer	1975	20.70

### Variable selection

**Fig. 3:**
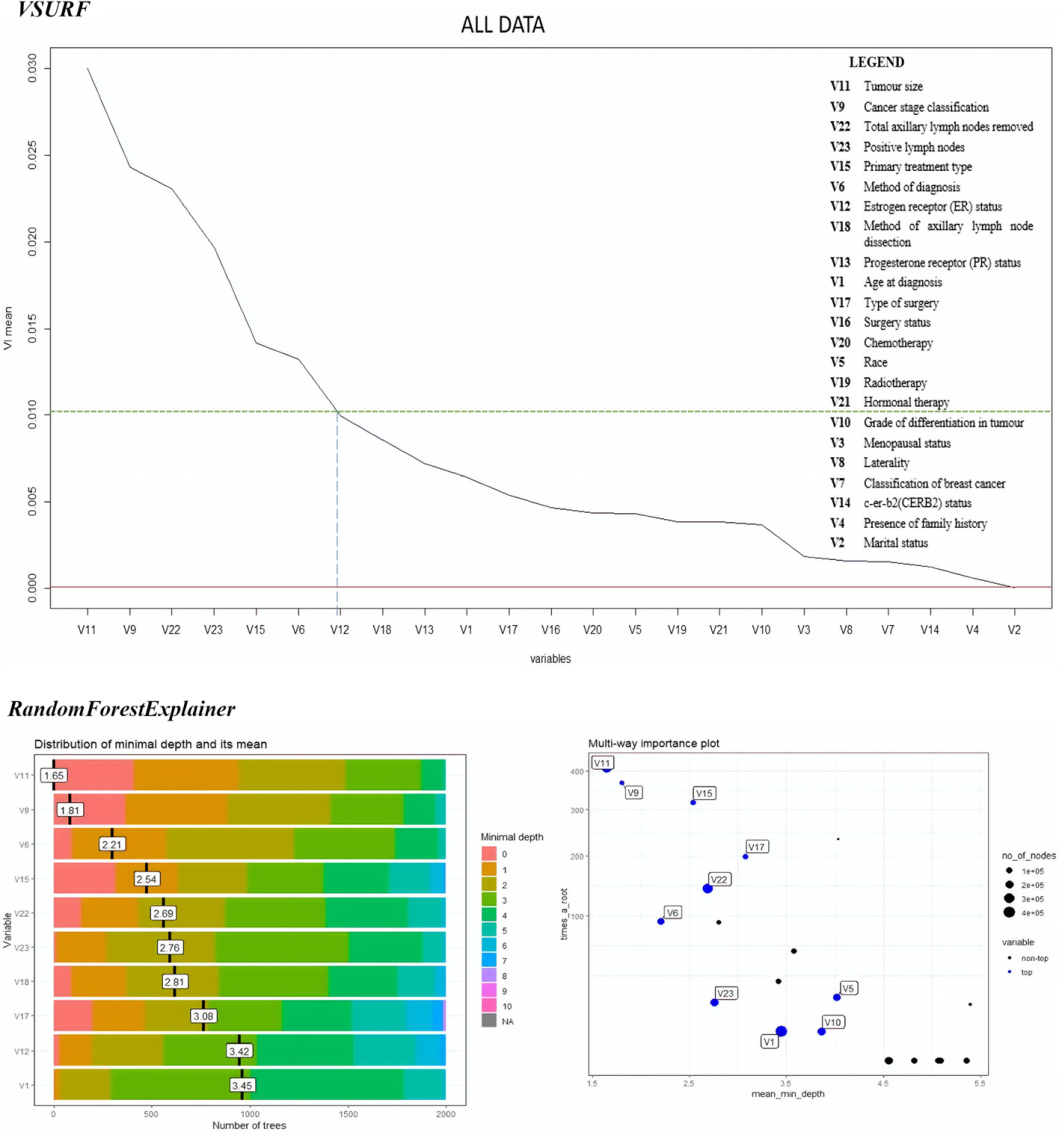
Variable importance plots of “All Data”

**Fig. 4:**
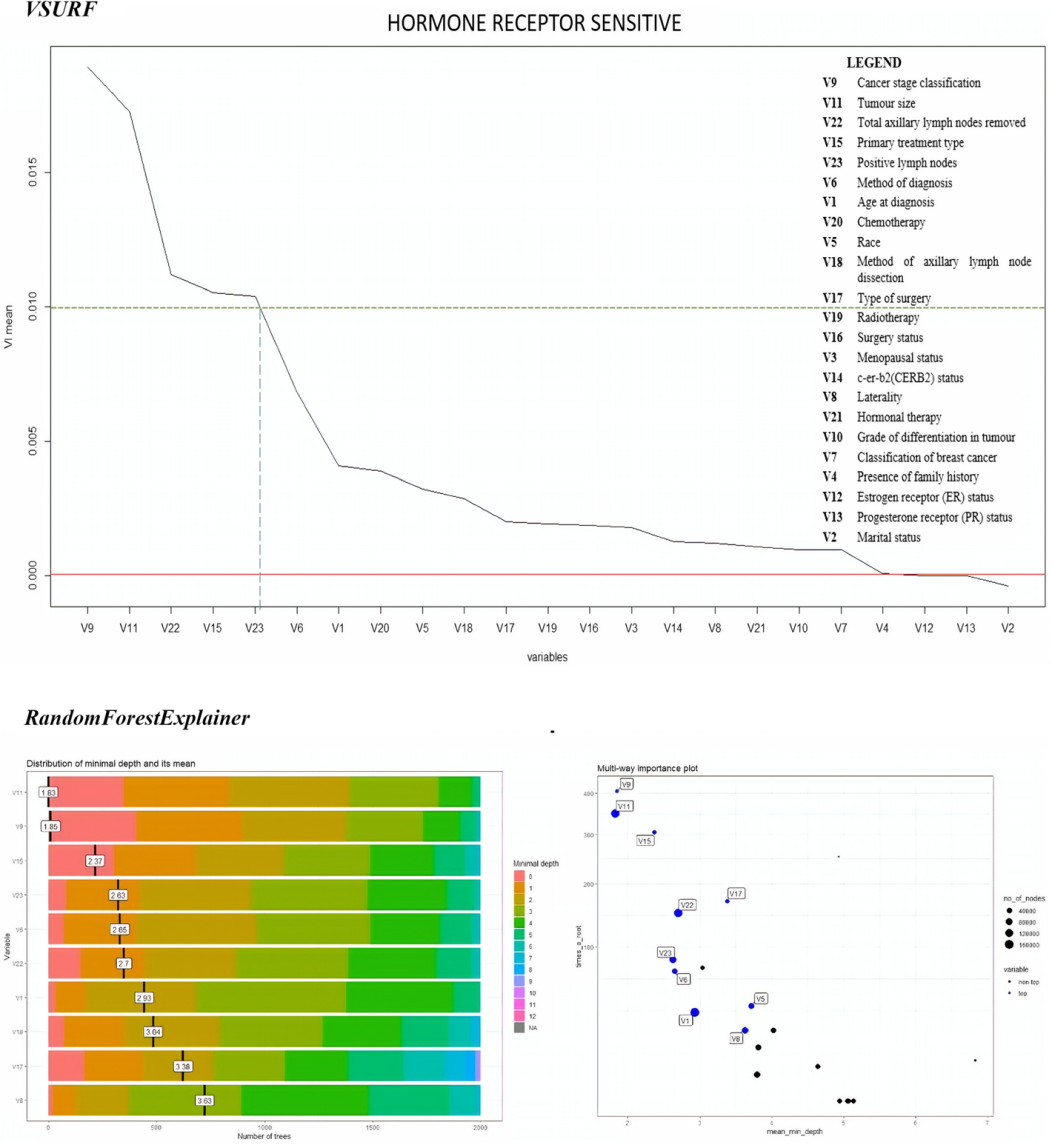
Variable Importance plots of “Hormone Receptor Sensitive” cluster

**Fig. 5:**
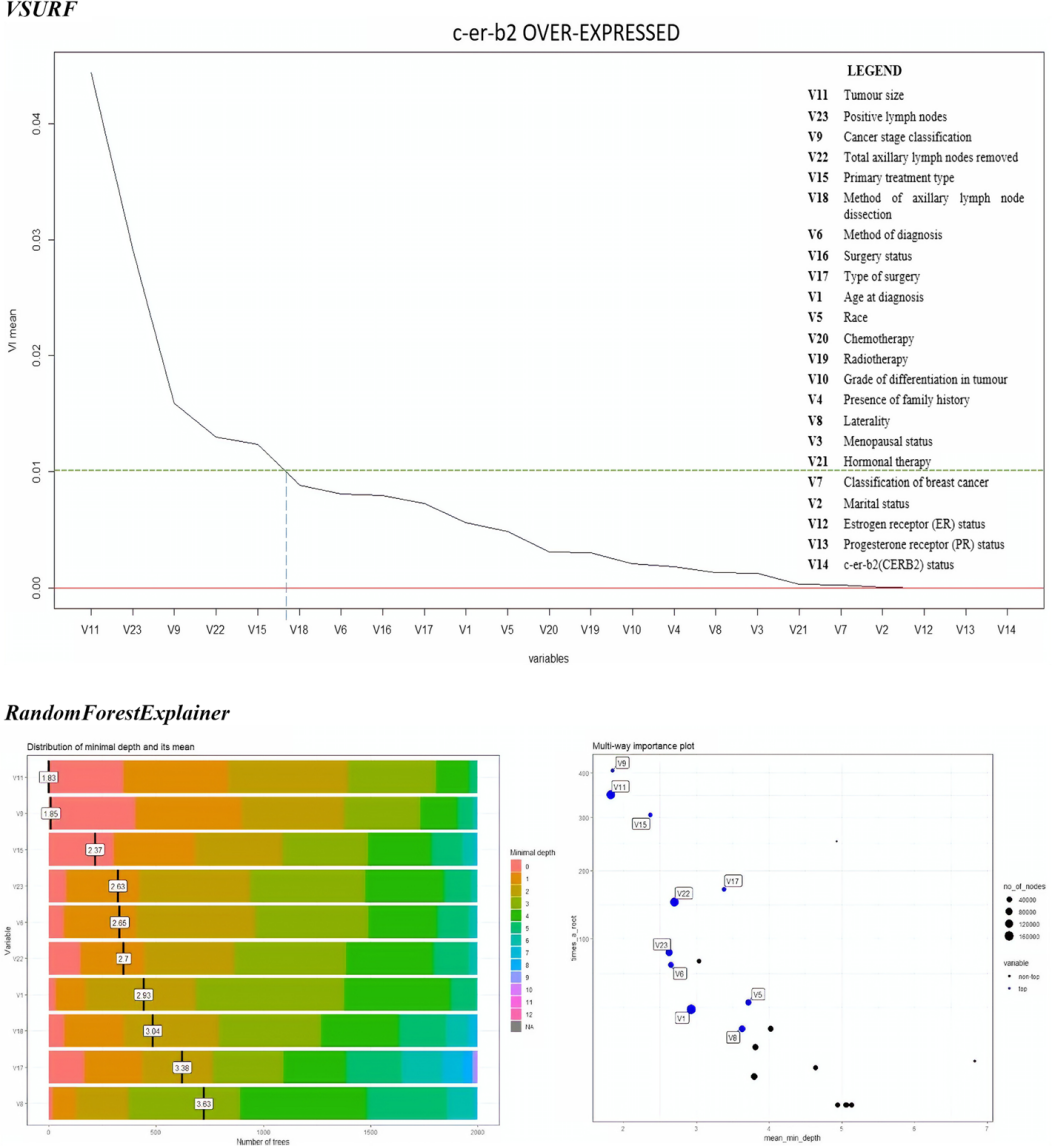
Variable Importance plots of “c-er-b2 over-expressed” cluster

**Fig. 6:**
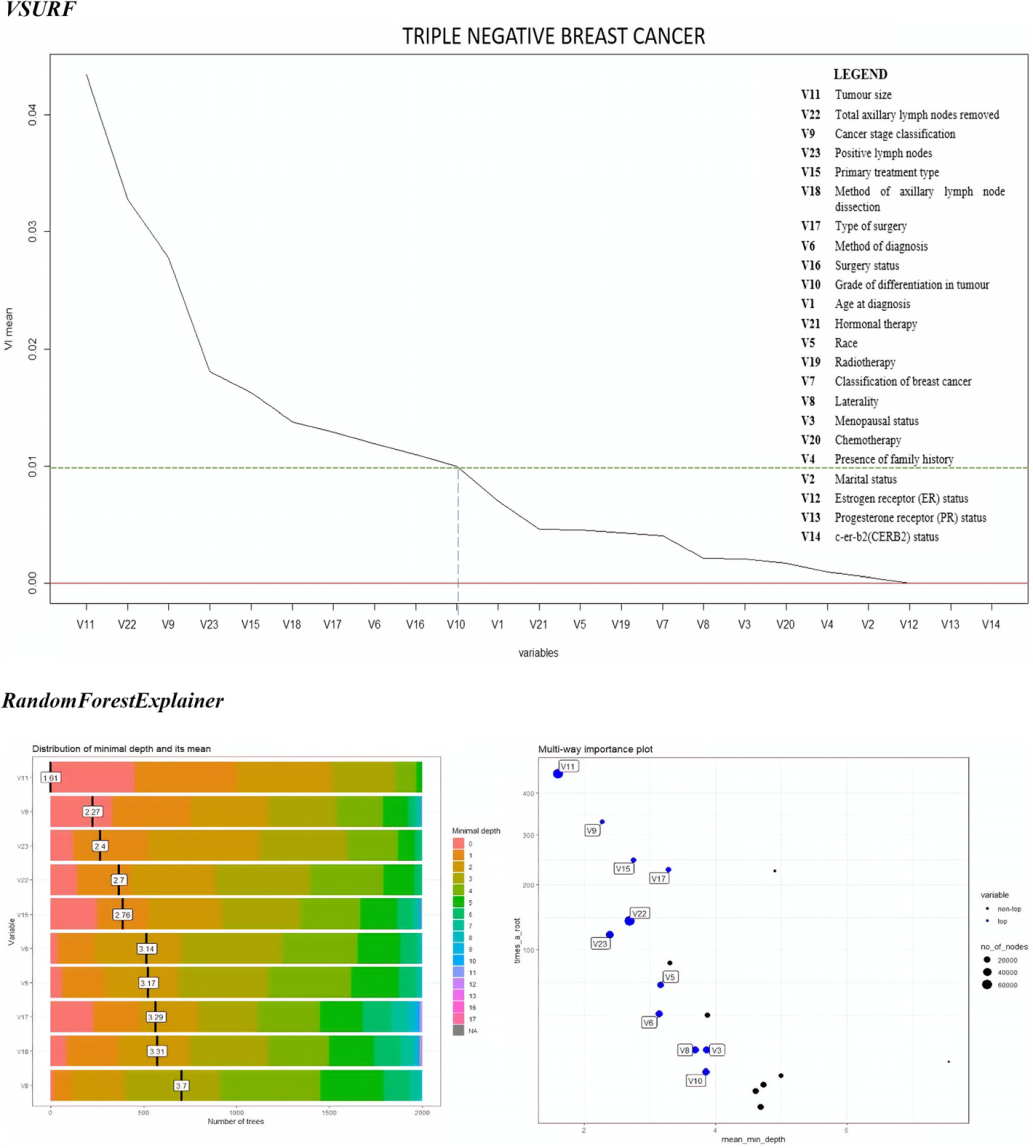
Variable Importance plots of “Triple Negative Breast Cancer” cluster

The results of variable selection are presented in Figs. 3, 4, 5 and 6. The figures display the comparison between the outputs produced by *VSURF* and *randomforestExplainer* packages. The mean of variable importance in the variable importance (VI) plot produced by the *VSURF* package measured the importance of each variable, where increment in VI mean indicated increased importance of the variable. The threshold value (VI mean) set to choose the most important variables was 0.01.

The *randomForestExplainer* package yielded two plots for each cluster, where one illustrated the important variables with their distribution of minimal depth and mean, while the second referred to the multi-way importance plot that classified the variables as the most important and less important variables. The range of distribution of minimal depth was between 0 and 10, where the importance of variable increased with decreasing mean values. The multi-way importance plot illustrates the most important variables in blue dots, whereas the less important variables in black dots. Six important variables were selected for each cluster to be compared with the variable importance plots produced by *VSURF* package.

The comparison between important prognostic factors of breast cancer survival identified by both *VSURF* and *randomForestExplainer* packages for all the data clusters is given in Table 6.

**Table 6:** Comparison of important prognostic factors of breast cancer survival rate

Cluster	*VSURF* (cut-off VI mean = 0.01)	*RandomForestExplainer* (First 6 variables)
All data	V11: Tumor size > V9: Cancer stage classification > V22: Total lymph nodes > V23: Positive lymph nodes > V15: Primary treatment type > V6: Method of diagnosis	V11: Tumor size > V9: Cancer stage > V6: Method of diagnosis > V15: Primary treatment type > V22: Total lymph nodes > V23: Positive lymph nodes
Hormone Receptor Sensitive (HRS)	V9: Cancer stage classification > V11: Tumor size > V22: Total lymph nodes > V15: Primary treatment type > V23: Positive lymph nodes	V11: Tumor size > V9: Cancer stage > V15: Primary treatment type > V23: Positive lymph nodes > V6: Method of diagnosis > V22: Total lymph nodes
CERB2 Over-expressed	V11: Tumor size > V23: Positive lymph nodes > V9: Cancer stage classification > V22: Total lymph nodes > V15: Primary treatment type	V11: Tumor size > V9: Cancer stage > V15: Primary treatment type > V23: Positive lymph nodes > V6: Method of diagnosis > V22: Total lymph nodes
Basal/Triple Negative Breast Cancer (TNBC)	V11: Tumor size > V22: Total lymph nodes > V9: Cancer stage classification > V23: Positive lymph nodes> V15: Primary treatment type > V18: Method of axillary lymph node dissection > V17: Type of surgery > V6: Method of diagnosis > V16: Surgery status	V11: Tumor size > V9: Cancer stage > stage > V23: Positive lymph nodes > V22: Total lymph nodes V15: Primary treatment type > V6: Method of diagnosis

### Decision tree

**Fig. 7:**
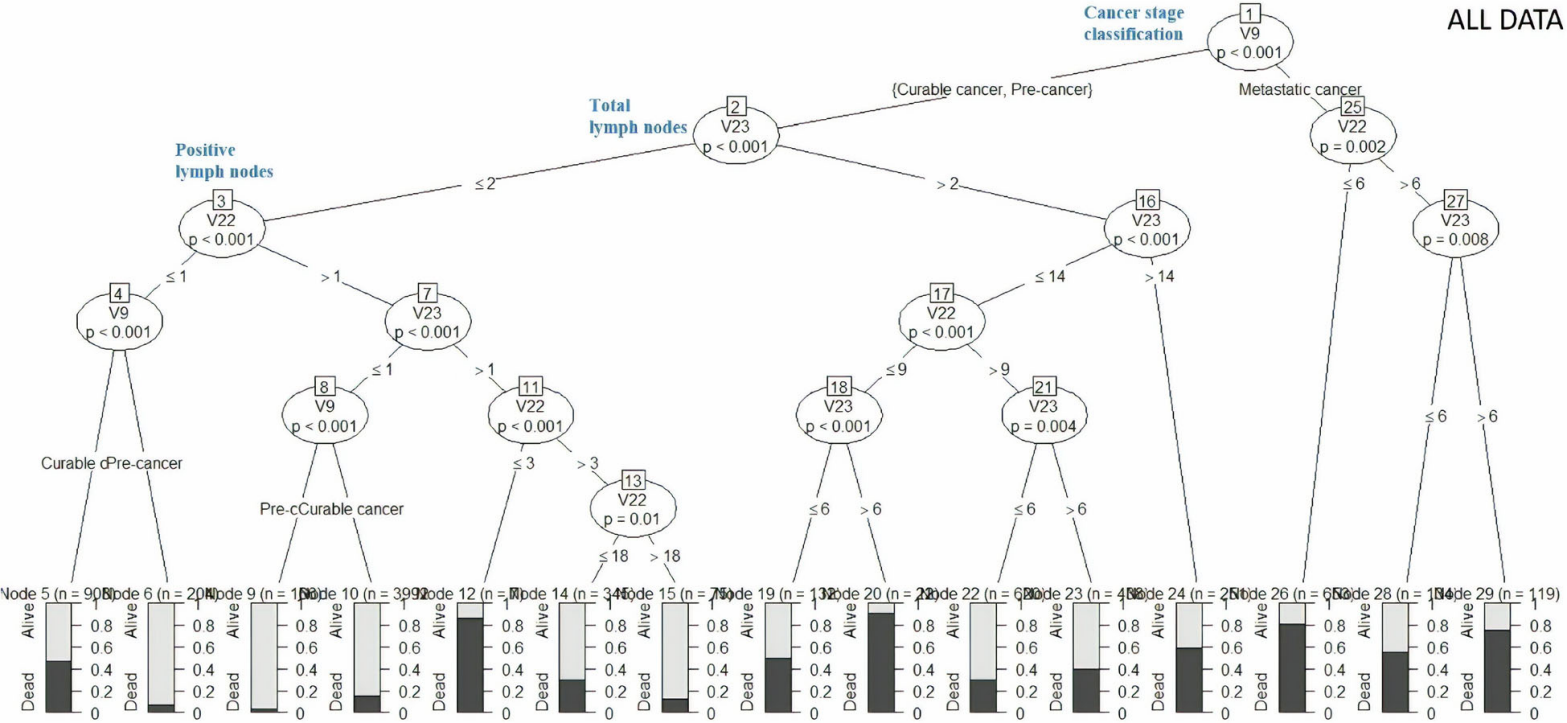
Decision tree for “All Data”

**Fig. 8:**
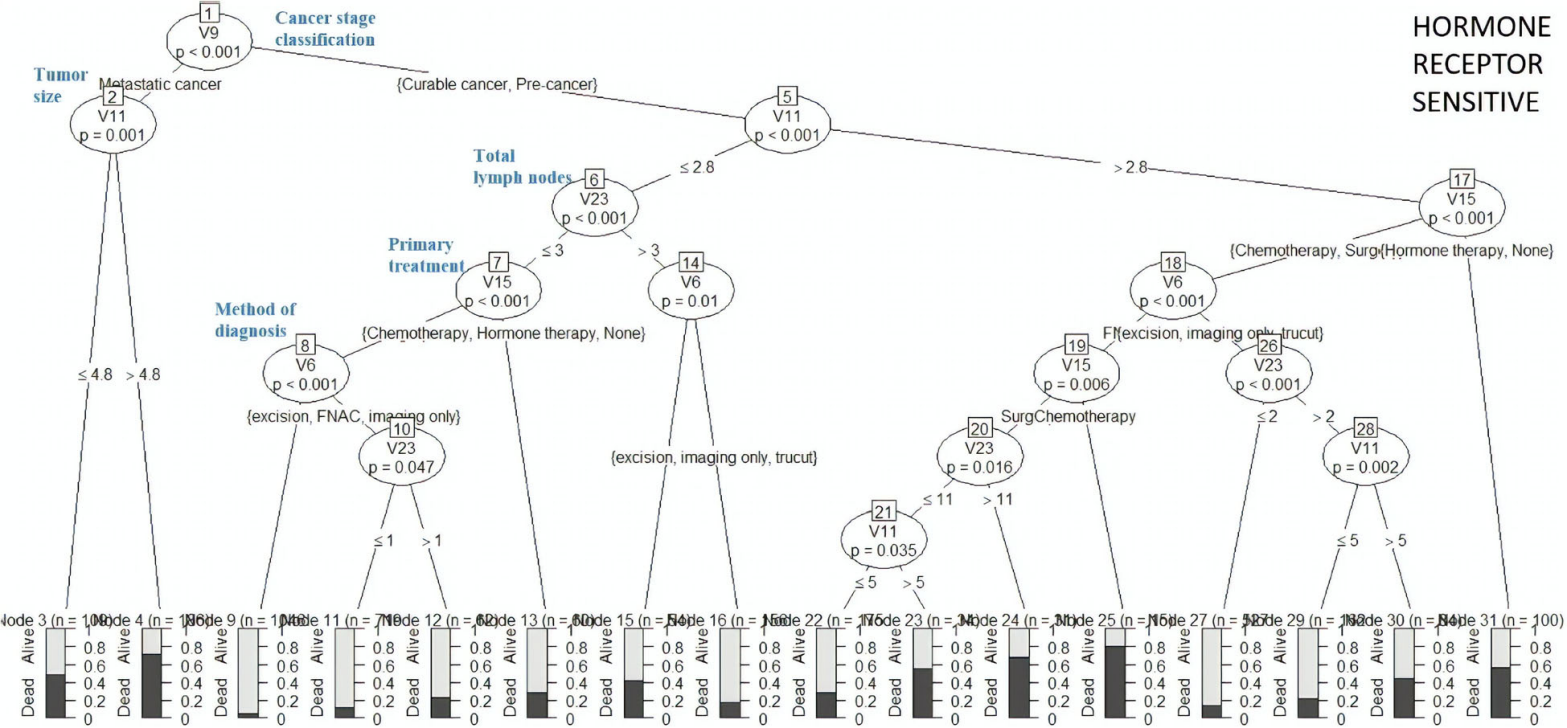
Decision tree for “Hormone Receptor Sensitive” cluster

**Fig. 9:**
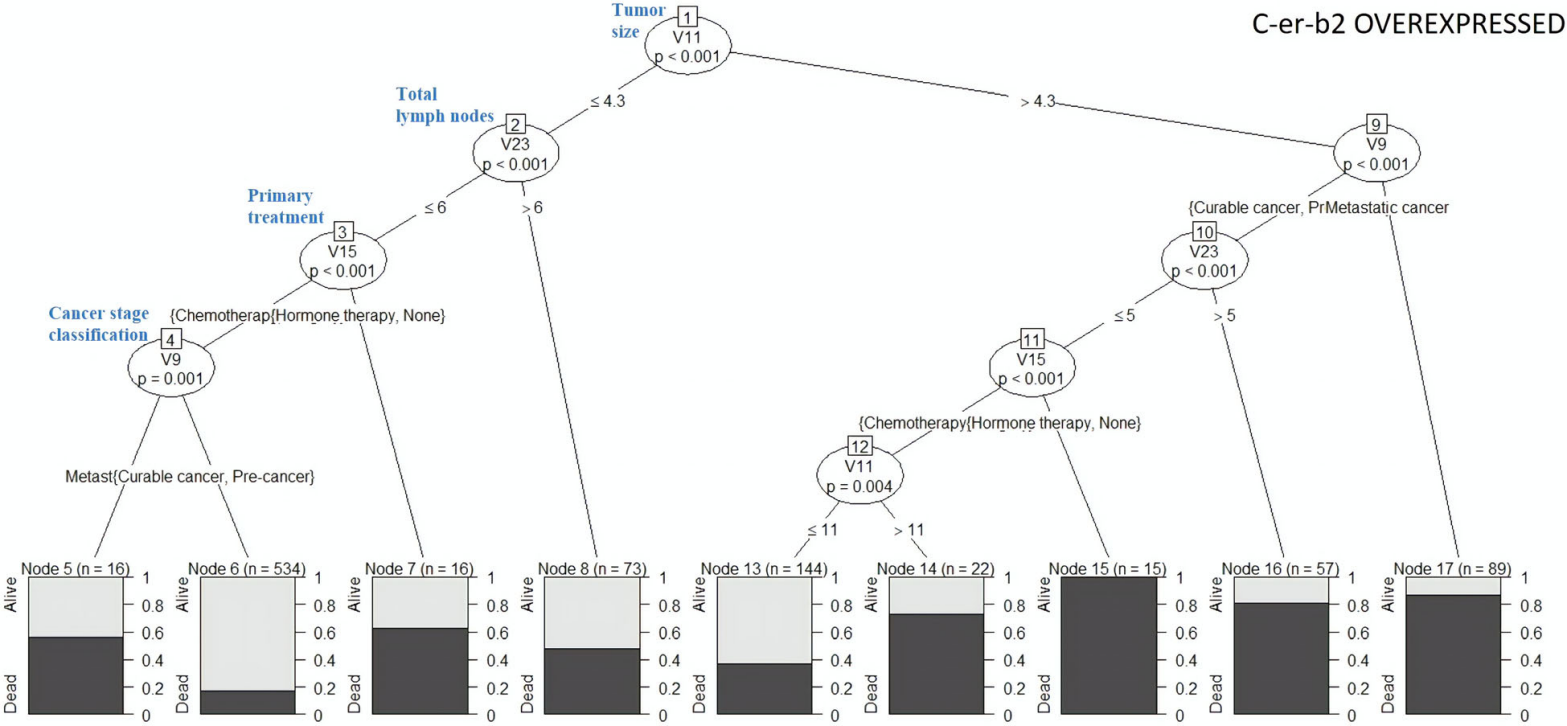
Decision tree for “c-er-b2 over-expressed” cluster

**Fig. 10:**
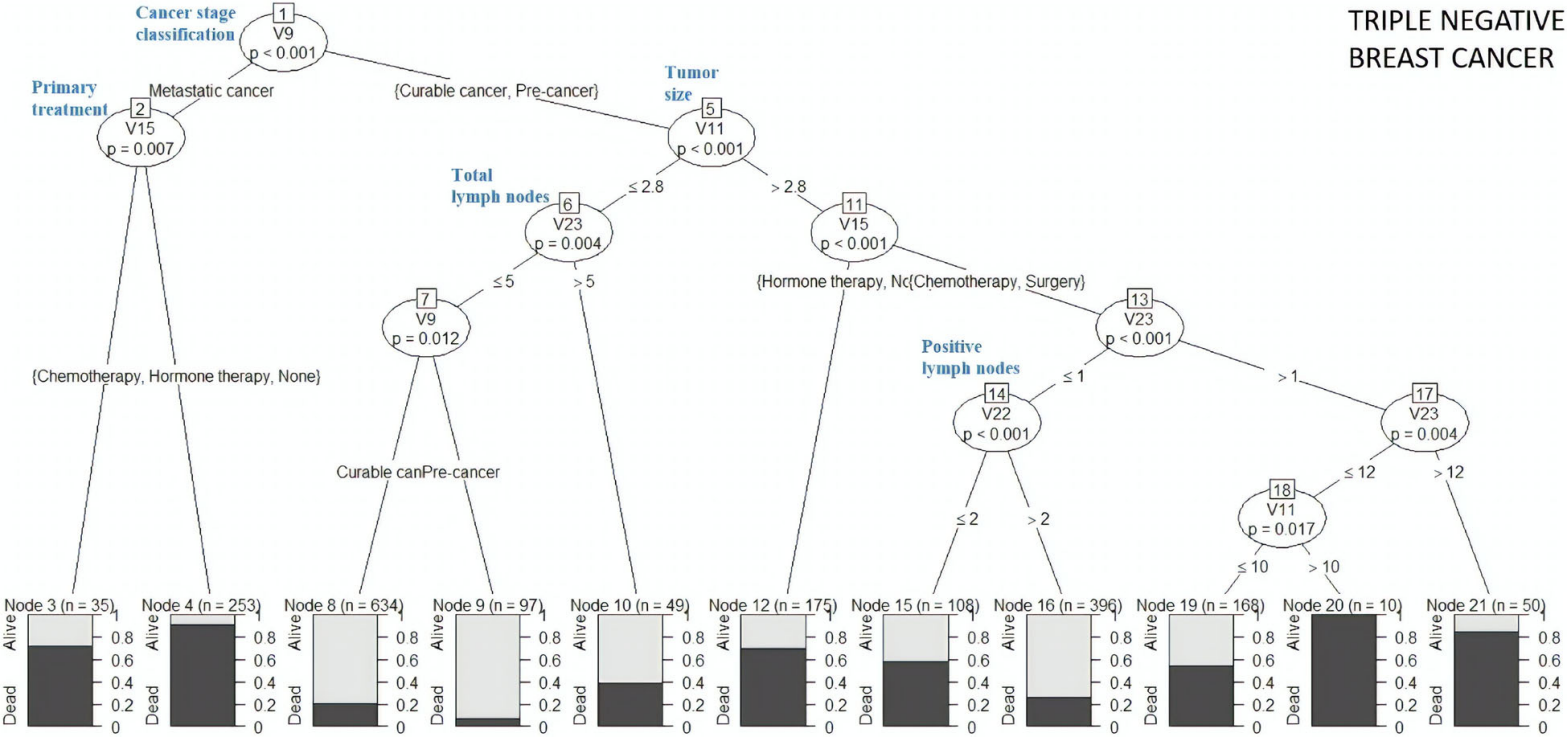
Decision tree for “Triple Negative Breast Cancer” cluster

The decision trees for ‘all data’ and three clusters are shown in Figs. 7, 8, 9 and 10.

Figure 7 shows that patients with curable cancer, ≤ 1 positive lymph nodes (PLN) and ≤ 2 total axillary lymph nodes removed (TLN) had 50% survival probability, while patients with pre-cancer, ≤ 1 PLN and ≤ 2 TLN had 90% survival probability. Patients with metastatic cancer, > 6 PLN and > 6 TLN had only 25% survival probability.

Figure 8 presents the DT for HRS cluster. Metastatic cancer patients with tumour size (TS) of ≤4.8 cm had a survival rate of 50%. Pre-cancer or curable cancer patients with TS ≤ 2.8 cm, TLN ≤ 3, surgery as primary treatment and diagnosed by excision, fine needle aspiration cytology (FNAC) or imaging methods had 90% survival rate.

Figure 9 illustrates that metastatic cancer patients with TS ≤ 4.3 cm, TLN ≤ 6, and chemotherapy as primary treatment had 40% survival rate. Patients with pre-cancer or curable cancer, TS ≤ 4.3 cm, TLN ≤ 6, and chemotherapy as primary treatment had 90% survival rate. Patients diagnosed with metastatic cancer and had TS > 4.3 cm indicated 10% of survival rate.

Figure 10 shows that metastatic cancer patients who were treated with chemotherapy or hormone therapy as primary treatment had 30% survival rate, while those who had undergone surgery as primary treatment had only 10% survival rate. Patients diagnosed with pre-cancer, having TS ≤ 2.8 cm and TLN ≤ 5 indicated 90% survival rate, while those with curable cancer had 80% survival rate.

### Survival analysis

**Fig. 11:**
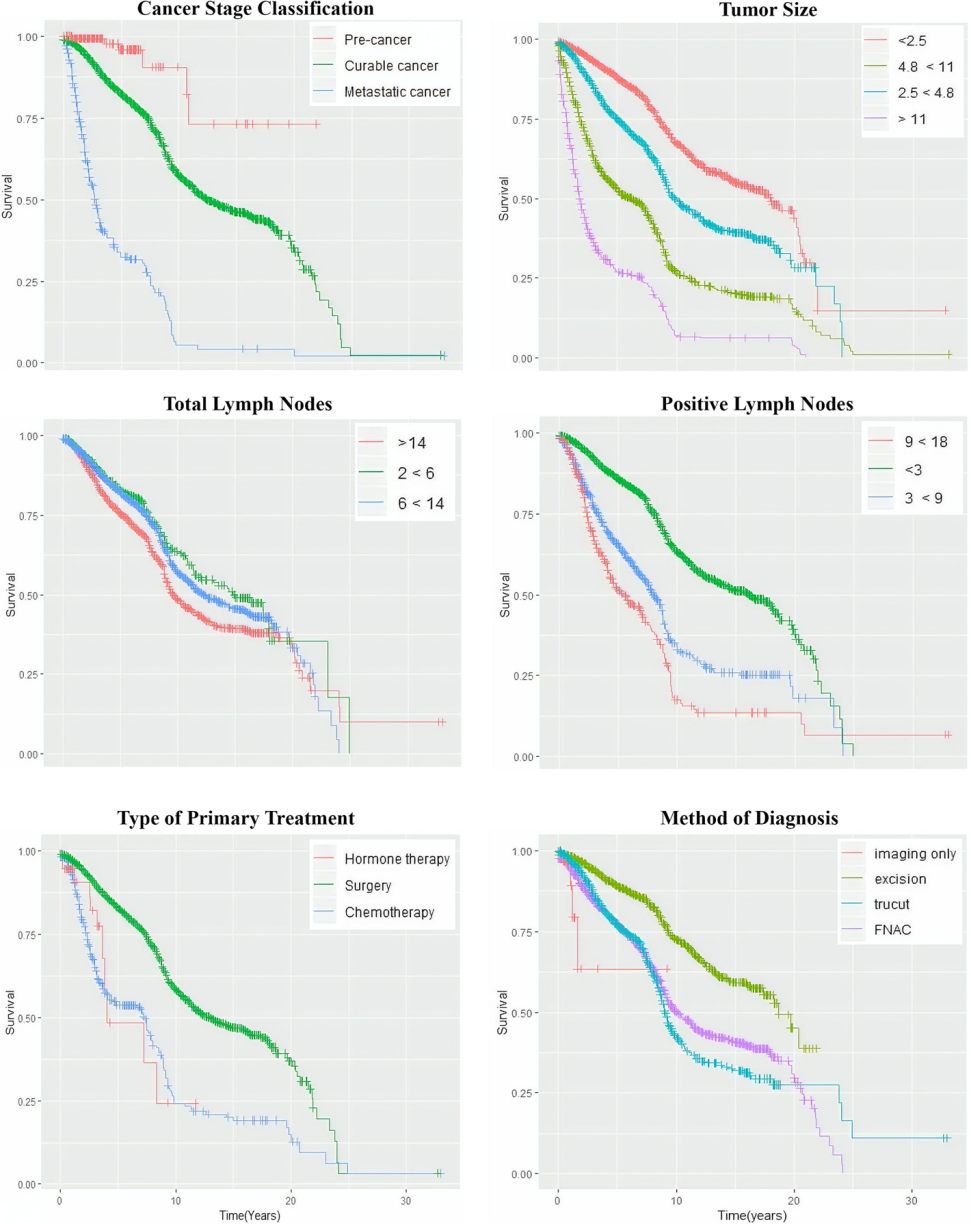
Survival curves of six important variables

The variables tabulated in Table 6 (cancer stage classification, tumour size (TS), total lymph nodes (TLN), positive lymph nodes (PLN), primary treatment, method of diagnosis) were used to produce the graphs illustrated in Fig. 11.

Pre-cancer patients displayed higher for 5 and 10 years of survival rates followed by curable and metastatic cancers. As for pre-cancer and metastatic cancer patients, their survival rate remained similar after 10 years, while for patients with curable cancer, a continuous decline was noted in their survival rate at the beginning and remained constant after 25 years.

The survival rate of the patients decreased as the range of TS exceeded from 2.5 cm to more than 11.0 cm. However, the survival rate began to remain constant for all patients after 20 to 25 years. A steep decrease was observed in the survival rate among patients with TS exceeding 11.0 cm, where they only had 25% of survival rate for 5 years.

The more the number of TLN removed, the lower the survival rate. The survival curves for TLN of 2 < 6 and TLN of 6 < 14 were almost close to each other until 10 years. The survival rate of patients with TLN > 14 began to drop drastically from the beginning and turned constant after 24 years, where the patients were closer towards reaching mortality.

The more the number of PLN identified, the lower the survival rate. Patients diagnosed with PLN between 9 and 18 had 50% survival rate for 5 years, and 15% survival rate for 10 years, which appeared constant after 10 years.

Those who underwent surgery had higher survival rate, when compared to those who were treated with chemotherapy and hormone therapies. The survival curves for patients with primary treatments of chemotherapy and hormone therapies intersected at 4 years of survival, where the survival rate was 55%. Patients treated with surgery had 50% survival rate for 10 years.

The excision method displayed higher survival rate, when compared to the other methods. “Fine needle aspiration cytology” (FNAC) and “trucut” methods gave similar survival rate until 10 years, and then, the FNAC exhibited slightly higher survival rate than the “trucut” method from 10 until 20 years. Nevertheless, the survival rate of patients diagnosed via “trucut” method remained constant after 20 years, while the FNAC method started to drop drastically after 20 years.

## Discussion

In this study, machine learning models were built using breast cancer data from the University Malaya Medical Centre to identify the important prognostic factors for breast cancer survival. All algorithms (decision tree, random forest, neural networks, extreme boost, logistic regression and support vector machine) yielded very close accuracies, with random forest being slightly higher. The calibration measures for all the six algorithms were also closer to the decision boundary. Variable selection performed on clusters of data based on receptor status using two different packages in random forest, reported similar variables as the most important prognostic factors of breast cancer survival. The decision trees and survival curves plotted for validation purpose, illustrated that the important variables identified are useful and could be used as a decision support tool by clinicians.

The top four important variables from the comparison between ‘all data’, ‘HRS’, ‘c-er-b2 over-expressed’, and ‘TNBC’ clusters were cancer stage classification, tumour size (TS), total axillary lymph nodes removed (TLN), and positive lymph nodes (PLN). These variables were also used in other studies for decision-making analysis in relation to breast cancer [50, 51]. Ture et al. [50], reported that TS and lymph node status were the best predictors in survival analysis of breast cancer. Besides, Sarvestani et al. [52], determined that the stage of cancer appeared to be the most important variable, followed by the number of positive nodes, and TS in a study using Surveillance, Epidemiology, and End Results (SEER) dataset, which consists of breast cancer patients’ data from the United States. Types of primary treatment was also among the top six factors predicted in this study. Similarly, the survival aspect exhibited by breast cancer patients was largely attributed to improvement in treatment administration in a Southeast Asia setting, thus pointing out the significance of treatment in predicting survival probability amongst those diagnosed with breast cancer [7]. Furthermore, surgery, a type of treatment, emerged as an important factor that may also be a surrogate to smaller operable tumours while the use of primary chemotherapy in this era is reserved to locally advanced breast cancer cases. The method of diagnosis was an essential prognostic factor in all four clusters. Tam et al. [53], stated that an early and accurate benign or malignant diagnosis of breast lesion should be determined pre-operatively prior to invasive surgery. The researchers in the same study also reported that a reliable pathological diagnosis aids in the planning of a definitive surgery, apart from minimising stress and burden among patients to manage treatment cost. Besides, the type of biopsy may indicate the biology of cancer, whether or not complete removal of the tumour has survival advantage from a needle core or FNAC. Thus, method of diagnosis plays an important role in estimating the survival rate of breast cancer patients. TLN appeared to be important, which is in line with other studies that reported the TLN as a significant prognostic factor of breast cancer [26, 54–56]. Grade of differentiation in tumour was not an essential feature in this study despite many studies, which used SEER dataset suggesting its role in prediction of breast cancer survival [57, 58]. The survival curves, as expected, highlighted the natural history of subtypes, where the survival of HRS continued to reduce after 15 years, whereas flattening mortality in TNBC and c-er-b2 clusters after 12 years. Further validation of important variables using the results of decision trees were compared with the American Joint Committee of Cancer (AJCC 5th edition) manual. Both TS and PLN are the determinants of the stage of breast cancer, according to AJCC and as expected, these were predicted as important variables in variable selection process in this study. The PLN size separation and TS were almost similar to N and T classifications in AJCC. The PLN separation generated from DT analysis of this study were (< 3, 3 < 9, and 9 < 18), whereas in the AJCC staging, PLN of less than or equals to 3 (N1) fell under Stage II breast cancer, PLN between 3 and 6 (N2) was categorised as Stage IIIA, and PLN exceeding 6 (N3) was under Stage IIIB. The AJCC system classifies the extent of disease based mostly on anatomic information on the extent of primary tumour, regional lymph nodes, and distant metastases. The TS separation in this study were (< 2.5 cm, 2.5 < 4.8 cm, 4.8 < 11 cm, and > 11 cm), while the AJCC manual categorised the TS as less than or equals to 2 cm (T1) for Stage I, 2–4 cm (T2) for Stage II, and more than 4 cm (T3) for Stage III.

In this study, only the clinical prognostic factors were used for prediction of survival rate, which could affect the overall and fair analysis of survivorship of patients. Hence, the decisions offered by the ML algorithms would be more comprehensive if both clinical and genomic data of breast cancer patients are analysed together. Additionally, the data used in this study is not representative of a complete Malaysian population as it is taken from a tertiary academic hospital situated in a relatively affluent part of the capital, which is preferred by predominantly middle class urban population [4]. In the future, this study can be extended to other public hospitals in Malaysia, in order to compare the outcome in women from different background, particularly from the rural area and the lower income group. The ultimate aim, nevertheless is to focus on other Asian regions where such studies are not carried out. Preferably, in the future, a combination of different algorithms can be implemented in evaluating model performance especially when using medical datasets.

The machine learning methods applied in this study can be translated into tools for clinical treatment decision-making, as many tools developed in the west do not fit into our population [59], for instance, the PREDICT tool that has been recently developed to enable the incorporation of novel prognostic biomarkers [60]. The visualisation of outcomes produced in this study will be implemented in the UMMC’s research database, *iResearch* to be used by the clinicians to analyse the survival of breast cancer patients administered at the hospital.

## Conclusion

This study presented analysis of prognostic factors of breast cancer survival using machine learning techniques. Model evaluation using random forest algorithm yielded slightly better accuracy when compared to other algorithms. Nevertheless, accuracies displayed by all the algorithms appeared close. The six most important variables identified in this study were cancer stage classification, tumour size, total axillary lymph nodes removed, positive lymph nodes, primary treatment type, and method of diagnosis. In the healthcare research, particularly using machine learning techniques, variable selection process may yield different results according to different dataset, location and lifestyle of patients. In this sense, this study has determined the model performance and important variables influencing survival rate of breast cancer patients, which can be employed in clinical practice, particularly within the Asian setting. Decision trees and survival curves built to validate the important variables that influence breast cancer survival rate in this study show that the visualisations of the results can be used to build predictive applications for survival analysis of various diseases within the medical domain.

## Abbreviations

DT: Decision tree
ER: Oestrogen receptor
HRS: Hormone receptor sensitive
IHC: Immunohistochemistry
ML: Machine Learning
OOB: Out-of-bag
PLN: Positive lymph nodes
PR: Progesterone receptor
RF: Random forest
SVM: Support vector machine
TLN: Total lymph nodes
TNBC: Triple negative breast cancer
TS: Tumour size
WEKA: Waikato environment for knowledge analysis
